# Adjuvant Chemotherapy in Children With Enucleated Retinoblastoma and Histopathologic High-Risk Features: Survival Outcomes From a Single Institution in a Middle-Income Country

**DOI:** 10.7759/cureus.99768

**Published:** 2025-12-21

**Authors:** Amaranto Suárez, Fernando Rojas, Jennifer Camargo, Javier Muñoz, Nathalie Soler, Maria Alejandra Ágredo Lucio, David Garay, Martha Piña

**Affiliations:** 1 Pediatric Oncology, Instituto Nacional de Cancerología, Bogota, COL; 2 Ophthalmic Oncology, Instituto Nacional de Cancerología, Bogota, COL; 3 Pediatric Oncology, Clínica Foscal, Bucaramanga, COL

**Keywords:** adjuvant chemotherapy, enucleation, event-free survival, extraocular relapse, histopathologic high-risk features, retinoblastoma, vincristine–etoposide–carboplatin

## Abstract

Background: Retinoblastoma is the most common intraocular malignancy in children. Although primary enucleation continues to be a standard treatment for advanced disease, specific histopathologic high-risk features substantially increase the likelihood of extraocular dissemination. Adjuvant chemotherapy aims to reduce this risk; however, evidence from middle-income countries remains limited.

Objective: To determine the prevalence of histopathologic high-risk features in enucleated retinoblastoma and to assess relapse risk and event-free survival (EFS) among patients treated with adjuvant chemotherapy.

Methods: We conducted a retrospective analytical study of children with unilateral retinoblastoma treated with primary enucleation and of those with bilateral disease in whom the more affected eye was enucleated before any therapy, between January 2008 and December 2018. Patients presenting high-risk histopathologic features received adjuvant chemotherapy with vincristine, etoposide, and carboplatin (VEC). EFS was estimated using the Kaplan-Meier method and compared using the log-rank test.

Results: A total of 101 patients were included; 74 (73%) had unilateral disease. The mean age at diagnosis was 2.1 years. Most eyes demonstrated advanced intraocular involvement (International Classification of Intraocular Retinoblastoma (ICRB) Group E: 82, 81.2%; Group D: 13, 12.9%). Histopathologic high-risk features were common: choroidal invasion occurred in 42 (41.6%) (17, 40.4% massive ≥3 mm), postlaminar optic nerve invasion in 11 (26.2%), and positive optic nerve margin in 16 (38%). Scleral invasion was identified in 20 (21.5%), and anterior chamber invasion in 14 (14%). Thirteen patients (13%) relapsed, all with fatal outcomes. Ten-year EFS was 83.3%. Massive choroidal invasion, scleral invasion, and postlaminar optic nerve invasion were the strongest predictors of poor outcome, and their coexistence was associated with the highest relapse risk.

Conclusions: Histopathologic high-risk features are common among children undergoing enucleation for advanced retinoblastoma. Adjuvant chemotherapy is warranted in cases with massive choroidal invasion, scleral infiltration, or postlaminar optic nerve involvement, given their strong association with metastatic relapse.

## Introduction

Retinoblastoma is the most common intraocular malignancy of childhood, accounting for approximately 3% of all childhood malignancies. Two-thirds of children with retinoblastoma are under two years of age at diagnosis, and 95% of cases are diagnosed before the age of 5 years.

Enucleation remains a definitive treatment option for children with unilateral intraocular retinoblastoma, achieving cure rates of approximately 90% [1-2]. In nearly 95% of cases, enucleation results in complete removal of the tumor; however, in high-income countries, fewer than 5% show microscopic residual disease after enucleation. In contrast, this figure is more variable in low- and middle-income countries (LMICs), where its prevalence may range between 23% and 78% [3]. In such settings, decisions regarding adjuvant chemotherapy to reduce the risk of metastatic disease should be based on the histopathologic evaluation of the enucleated eyes.

The study aimed to present the outcomes of the ACHOP 2007 protocol (Asociación Colombiana de Hemato-Oncología Pediátrica) to assess survival in patients with risk factors for developing metastatic disease, based on the histopathologic characteristics of enucleated eyes, who were treated at the National Cancer Institute of Colombia with adjuvant chemotherapy using the VEC regimen (Vincristine, Etoposide, and Carboplatin).

## Materials and methods

We conducted an analytical observational study of a historical cohort with survival analysis, including patients younger than 15 years, both sexes, treated between January 1, 2008, and December 31, 2018, who had either unilateral enucleation or bilateral disease in which the more advanced eye was enucleated before any treatment. All patients had a confirmed diagnosis of intraocular retinoblastoma based on pathology, were free of metastatic disease (International Retinoblastoma Staging System (IRSS) Stage II), and had not received prior chemotherapy, laser thermotherapy, or radiotherapy. Follow-up information was updated through July 31, 2022.

All medical records of patients coded as C69.2 under ICD-10 were reviewed. Upon admission, all patients underwent an ophthalmologic evaluation that included ocular ultrasound, bilateral fundus examination under general anesthesia with maximal pupillary dilation, and indirect ophthalmoscopy. Additionally, computed tomography (CT) or magnetic resonance imaging (MRI) of the orbits and brain was performed. Intraocular tumors were classified using the International Classification of Intraocular Retinoblastoma (ICRB), and extra-retinal disease was staged using the IRSS, as established by Shields et al. and Chantada et al., respectively [4,5].

Histopathologic involvement of enucleated eyes was assessed by the institutional pathology department. High-risk histopathologic features were defined as involvement of one or more of the following structures: massive choroidal invasion, sclera, or optic nerve.

Definitions of choroidal, scleral, and optic nerve involvement followed the recommendations of the International Retinoblastoma Staging Working Group (IRSWG) consensus on histopathologic risk factors for extraocular relapse [6], complemented by the IRSWG pathological staging criteria for intraocular tumors with or without high-risk features and for extraocular extension, including assessment of the optic nerve section margin [7].

Choroidal Involvement

Focal choroidal involvement is defined as a tumor focus <3 mm in any dimension (thickness or width) that does not reach the sclera. Massive choroidal involvement (posterior uveal invasion) is defined as choroidal invasion ≥3 mm in maximum dimension (thickness or width); multiple foci of focal choroidal invasion totaling >3 mm in any dimension; or any full-thickness choroidal invasion. Most tumors meeting this definition extend to at least the inner scleral fibers.

Scleral Involvement

Defined as any partial-thickness invasion of the sclera within the inner two-thirds, full-thickness invasion into the outer one-third, or invasion within or surrounding the emissary canals.

Optic Nerve Involvement

Local invasion is defined as the presence of tumor cells within the prelaminar or intralaminar region of the optic nerve head. Significant local invasion is defined as postlaminar optic nerve invasion (PLONI) without involvement of the optic nerve section margin, involvement of the section margin, or the presence of tumor within the meningeal spaces surrounding the optic nerve.

Due to the retrospective nature of the study, there was no blinding of the pathologists, and in cases where the diagnosis of the histopathological characteristics presented any difficulty in the Institution, paired reviews were performed for these cases.

All patients underwent renal and liver function testing, serum electrolyte measurement, and complete blood counts before initiation of chemotherapy and before each treatment cycle.

Patients with high-risk histopathologic features (massive choroidal invasion, scleral invasion, PLONI, or anterior chamber invasion) received six cycles of adjuvant chemotherapy with the VEC regimen, consisting of vincristine 0.05 mg/kg in children <36 months or 1.5 mg/m² in those ≥36 months on day 1; etoposide 5 mg/kg in children <36 months or 150 mg/m² in those ≥36 months on days 1 and 2; and carboplatin 18.6 mg/kg in children <36 months or 560 mg/m² in those ≥36 months. Treatment cycles were administered every 21-28 days, depending on hematologic recovery, renal function, and electrolyte normalization.

Time-to-relapse or death was analyzed using survival function estimation by the Kaplan-Meier method. Comparisons of survival were performed using the log-rank test.

The primary outcome was event-free survival (EFS), defined as the time from diagnosis to the occurrence of the first event (relapse, death, or second malignancy) or last follow-up for event-free patients. The secondary outcome was time to death, overall survival (OS), defined as the time from diagnosis to death from any cause, or last follow-up. Patients without events were censored at the date of last follow-up. Survival functions were estimated using the Kaplan-Meier method, and survival comparisons were performed using the log-rank test. Statistical analyses were two-tailed with a type I error rate of 0.05.

The study was reviewed and approved by the Ethics and Research Committee of the National Cancer Institute of Colombia.

## Results

A total of 101 patients diagnosed with retinoblastoma were evaluated; 74 (73%) had unilateral disease, and 27 (27%) had bilateral disease.

The mean age at diagnosis was 2.1 years (SD ±1.64), with a similar distribution by sex. The most common presenting symptoms were leukocoria (93, 93%) and strabismus (40, 40%). Most patients presented with advanced intraocular disease, with 82 (81.2%) classified as Group E and 13 (12.9%) as Group D according to the International Classification of Intraocular Retinoblastoma (ICRB).

Histopathologic examination of the enucleated eyes revealed involvement of the following structures:

Choroidal invasion: Forty-two patients (41.6%) had choroidal invasion; among them, 17 (40.4%) exhibited massive invasion (≥3 mm), and 25 (59.5%) had focal invasion (<3 mm).

Optic nerve invasion: Forty-two patients (41.6%) showed optic nerve involvement; 15 (35.7%) had prelaminar invasion, 11 (26.2%) had postlaminar invasion, and 16 (38%) had tumor involvement at the optic nerve section margin.

Scleral involvement: Twenty eyes (21.5%) demonstrated scleral involvement, though only 4 (20%) exhibited isolated scleral invasion. One patient had massive choroidal involvement; three had postlaminar optic nerve involvement (PLONI); three had involvement of the optic nerve section margin; six had both massive choroidal invasion and margin involvement; two had massive choroidal invasion with prelaminar optic nerve involvement; and one had massive choroidal invasion with postlaminar invasion but without margin involvement.

Anterior chamber involvement: Fourteen patients (14%) showed tumor invasion of the anterior chamber. Table 1 summarizes demographic and clinical characteristics.

**Table 1:** Demographic and clinical characteristics of 101 patients with retinoblastoma undergoing primary enucleation. ICRB, International Classification of Intraocular Retinoblastoma

Demographic and clinical characteristics	*n* (%) (*n* = 101)
Mean age in years (SD)	2.14 (± 1.64)
Male sex n (%)	45 (44.6)
Symptoms at diagnosis n (%)	
Leukocoria	93 (93)
Strabismus	40 (40)
Red eye	25 (25)
Buphthalmos	24 (24)
Glaucoma	4 (4)
Laterality, *n* (%)	101 (100)
Unilateral	74 (73)
Bilateral	27 (27)
Vitreous seeding, *n* (%)	68 (68)
Anterior chamber involvement, *n* (%)	14 (14)
Intraocular ICRB classification, *n* (%)	98 (97)
A	1 (1)
B	1 (1)
C	1 (1)
D	13 (12.9)
E	82 (81.2)
Enucleated, *n* (%)	93 (93)
Exenterated, *n* (%)	8 (8)
Choroidal involvement, *n* (%)	42 (41.6)
Focal	25 (59.5)
Massive	17 (40.4)
Scleral involvement, *n* (%)	20 (21.5)
Optic nerve involvement	42 (41.6)
Prelaminar	15 (35.7)
Postlaminar	11 (26.2)
Transection margin	16 (38.0)

Thirteen patients (13%) relapsed, with bone marrow and central nervous system involvement being the most frequent sites, either isolated or combined. Two patients developed local orbital relapse. All patients who relapsed died. The cumulative incidence of death was 14 (14%), with most deaths occurring secondary to relapse and disease progression. There were no deaths due to neutropenia associated with treatment toxicity; however, one patient died as a result of sepsis without neutropenia. No patient developed a second malignancy. Event distribution is presented in Table 2.

**Table 2:** Number of events (relapses and deaths) in 101 patients with enucleated retinoblastoma. CSF, cerebrospinal fluid

Relapses, *n* (%)	Value
Types of relapses, *n* (%)	13 (13)
Isolated CSF	2 (15.3)
Bone marrow	2 (15.3)
Orbit	2 (15.3)
Bone marrow and CSF	3 (23.0)
Bone marrow and bone	4 (30.6)
Deaths, *n* (%)	14 (14)
Causes of death	
Relapse and progression	13 (93)
Sepsis without neutropenia	1 (7)

The bivariate analysis evaluating relapse risk for each high-risk histopathologic factor showed that massive choroidal invasion conferred a higher risk of relapse (relative risk (RR) 9.545, 95% confidence interval (CI) 2.317-38.328, *P* = 0.002). Likewise, scleral invasion (RR 22.667, 95% CI 4.283-119.999, *P* = 0.0001) and tumor involvement at the optic nerve section margin (RR 2.246, 95% CI 1.287-3.919, *P* = 0.0001) were statistically significant predictors of relapse. Focal choroidal invasion and pre-laminar optic nerve involvement or PLONI with a negative section margin were not associated with increased relapse risk (Table 3).

**Table 3:** Risk of relapse in patients with enucleated retinoblastoma according to histopathological high-risk factors. RR, relative risk; CI, confidence interval

	n	Relapses	No relapses	RR	95% CI	P
Absence of focal choroidal invasion	67	9	58			
Presence of focal choroidal invasion	25	1	24	0.269	0.032-2.237	0.276
Absence of massive choroidal invasion	74	4	70			
Presence of massive choroidal invasion	17	6	11	9.545	2.317-39.328	0.002
Negative scleral invasion	70	2	68			
Positive scleral invasion	20	8	12	22.667	4.283-119.999	0.0001
Negative anterior chamber involvement	72	4	68			
Positive anterior chamber involvement	14	7	7	17	3.971-72.771	0.0001
Negative prelaminar optic nerve involvement	58	1	57			
Positive prelaminar optic nerve involvement	15	2	13	0.114	0.110-1.355	0.105
Negative postlaminar optic nerve involvement	58	1	57			
Positive postlaminar optic nerve involvement	11	1	10	0.175	0.010-3.039	0.295
Negative optic nerve section margin	58	1	57			
Positive optic nerve section margin	16	9	7	2.246	1.287-3.919	0.0001

EFS with a mean follow-up of 10 years (95% CI 10.89-12.88) for the entire cohort of enucleated retinoblastomas was 83%. Among the 54 patients (53.5%) with at least one high-risk histopathologic factor who therefore received adjuvant chemotherapy, EFS was 76%. In contrast, the 47 patients (46.5%) without high-risk features and who did not receive adjuvant chemotherapy had an EFS of 93.3%.

Massive choroidal invasion resulted in an EFS of 60.7% compared with 93.4% in patients without massive invasion (*P* = 0.001). Scleral invasion was associated with a six-year EFS of 46.8% versus 96% in patients without scleral involvement (*P* = 0.000). None of the patients presented isolated postlaminar optic nerve invasion (combinations included massive choroidal and scleral invasion: 2 patients; massive choroidal invasion and anterior chamber involvement: 1; scleral invasion alone: 5; anterior chamber plus scleral invasion: 2; and massive choroidal with anterior chamber and scleral invasion: 1).

The presence of a positive optic nerve section margin had the greatest impact on EFS, with a 3.6-year EFS of 28.8% compared with 96.2% for those without margin involvement (*P* = 0.000). Table 4 summarizes EFS and OS by high-risk histopathologic feature.

**Table 4:** Event-free survival (EFS) and overall survival (OS) according to histopathological high-risk factors in children with enucleated retinoblastoma.

Risk factors	EFS positive	EFS negative	Log-rank	OS positive	OS negative	Log-rank
Focal choroid	0.950	0.840	0.231	0.950	0.843	0.246
Massive choroid	0.600	0.930	0.000	0.600	0.933	0.000
Prelaminar optic nerve	0.836	0.963	0.186	0.836	0.962	0.260
Postlaminar optic nerve	0.909	0.963	0.411	0.900	0.962	0.402
Sclera	0.468	0.967	0.001	0.465	0.966	0.001
Anterior chamber	0.490	0.914	0.001	0.490	0.910	0.001
Positive optic nerve section margin	0.324	0.930	0.001	0.27.5	0.962	0.001

## Discussion

Retinoblastoma is a malignant neuroblastic tumor arising from retinal photoreceptor cells. It is the most common intraocular malignancy of childhood, with an incidence of 1 per 15,000-20,000 live births. Its standardized incidence rate among children under five years varies widely, ranging from 3 to 5 cases per million person-years in high-income countries to 6 to 10 per million in LMICs [3].

The disease has favorable outcomes when the tumor remains intraocular; however, once extraocular extension occurs, the result is generally poor, even with the use of chemotherapy and radiotherapy. Survival for children with non-metastatic retinoblastoma reaches 90%-95% in high-income countries but remains below 50% in LMICs [8].

Although conservative treatment of intraocular retinoblastoma to preserve the eye, initially systemic chemotherapy followed by focal therapy, has changed in recent years with the use of intra-arterial and intravitreal chemotherapy in high- and upper-middle-income countries, enucleation remains necessary for patients with advanced intraocular disease. It continues to yield cure rates of up to 90%. Nevertheless, some patients experience extraocular relapse, with a probability of death between 44% and 60%, particularly when the central nervous system is involved [9].

International consensus groups agree that certain histopathologic characteristics identified in enucleated eyes constitute risk factors for relapse and death. These findings justify the use of adjuvant chemotherapy after enucleation in patients considered at high risk for extraocular progression.

Consistent with findings from other groups, our study showed no statistically significant differences in outcomes associated with focal choroidal infiltration or prelaminar optic nerve involvement. These features should therefore be considered low-risk, and patients presenting only these findings may be managed with enucleation alone, without adjuvant chemotherapy or radiotherapy [10-12]. In contrast, massive choroidal invasion was strongly associated with increased relapse risk (RR 17.4; 95% CI 2.075-146.072; *P* = 0.004) and with markedly reduced EFS (60.7% vs. 93.7%; *P* = 0.001). All patients with isolated massive choroidal invasion or massive invasion combined with other high-risk features received adjuvant chemotherapy.

Although most retinoblastoma treatment groups have published in non-randomized studies that massive choroidal invasion is a high-risk histopathologic factor, the multicenter study by the Latin American Pediatric Oncology Group (GALOP) reported 100% EFS in 13 patients with isolated massive choroidal invasion who did not receive adjuvant chemotherapy [11]. Similarly, the Children's Oncology Group (COG), in a prospective study of metastatic risk factors, suggested that isolated choroidal invasion of any degree may not independently justify adjuvant chemotherapy, emphasizing that salvage chemotherapy at relapse can still achieve long-term survival [12]. However, the COG also acknowledged that relapse management typically requires intensive chemotherapy, with significant short- and long-term toxicities [13]. Based on these considerations, we propose that isolated massive choroidal invasion in LMICs, where hematopoietic stem cell transplantation is unavailable, be classified as an intermediate-risk group, in which reducing rather than eliminating adjuvant chemotherapy should be considered.

Interpretation of isolated PLONI was challenging in our cohort because no patient presented with postlaminar invasion in the absence of other high-risk features, and bivariate analysis of this feature did not reach statistical significance. Consequently, all patients received six cycles of adjuvant chemotherapy due to the presence of other concomitant high-risk histopathologic factors that may increase the risk of extra-medullary relapse. Other studies have shown that patients with isolated postlaminar involvement who received adjuvant chemotherapy had no relapses [10] or only a low cumulative incidence of relapse [9,11,14,15]. In our study, patients with postlaminar optic nerve invasion demonstrated no statistically significant difference in EFS compared with those without such involvement (90.9% vs. 96.3%; *P* = 0.411).

A high-impact factor in the survival of our cohort was the presence of a positive optic nerve section margin, with an EFS of 32.4% compared with 96.3% for those without margin involvement (*P* = 0.0001). Although margin involvement corresponds to extraocular disease (IRSS Stage II), we highlight this finding for two reasons. First, our results differ markedly from those of Künkele et al. [16], who reported an 80% survival rate using the same chemotherapy regimen combined with radiotherapy. Second, the finding reflects the reality of many LMICs, where late diagnosis is common. This is reflected in our series, where 81% of patients presented with Group E tumors and 13% with Group D tumors (ICRB), and among the 42 patients with optic nerve involvement, 16 (38%) had margin invasion. We believe that, in our setting, the higher proportion of advanced-stage disease and poorer EFS outcomes are associated with prolonged diagnostic delays, as previously reported in Central American countries by Luna-Fineman et al. in the AHOPCA II protocol [17], and by Canturk et al. in their review of socioeconomic factors influencing retinoblastoma survival [18].

Isolated scleral involvement occurred in only 4 of 101 patients (4%). All received adjuvant chemotherapy, and none relapsed. Meanwhile, 16 of the 20 patients with scleral invasion had combinations with other high-risk histopathologic factors. This significantly affected EFS, which was 51.7% in patients with scleral invasion compared with 96.7% in those without (*P* = 0.0001). Scleral invasion typically reflects advanced disease and is frequently associated with other high-risk findings, such as postlaminar optic nerve invasion > 1.5 mm and massive choroidal invasion [19], as observed in our cohort.

Anterior chamber invasion was identified in 14% of patients and was associated with significantly worse EFS (49% vs. 91.4%; *P* = 0.0001). However, no patient had isolated anterior chamber involvement, making it impossible to evaluate its independent prognostic significance. All cases occurred in combination with other high-risk features and therefore received adjuvant chemotherapy. Additionally, there was no clear definition that included invasion of the ciliary body, trabecular meshwork, or iris (anterior segment). These challenges in determining whether anterior chamber invasion alone represents a high-risk factor have also been described in other studies [12,20-22]. We believe that in countries with limited resources, with a high incidence of advanced intraocular disease and no possibility of centralized review by pathologists experienced in retinoblastoma histopathology, and without clear definitions of anterior segment involvement, adjuvant chemotherapy should continue to be indicated whenever the anterior chamber is involved.

We acknowledge that the retrospective design represents a significant limitation of this study, as it implies potential information biases, including incomplete histopathological reports, variability in the interpretation of involvement of some structures at high risk of extraocular recurrence, and inconsistent definitions of the histopathological risk categories.

## Conclusions

This study provides valuable insight into the disease burden and histopathologic risk profile of patients with retinoblastoma treated at a cancer center in a middle-income country. As seen in other low-resource settings, patients frequently present with advanced stages of disease and with high-risk histopathologic features that predispose them to increased risks of relapse and mortality. Massive choroidal invasion, scleral invasion, and PLONI were associated with the poorest outcomes. The presence of these concomitant factors warrants the use of adjuvant chemotherapy. The authors caution that the need for adjuvant chemotherapy for PLONI could not be supported by our data, but is supported by reports from other international groups

Finally, we must consider the retrospective nature of the study to exercise caution in decision-making based on a non-randomized, single-center study of children with retinoblastoma.
